# SUN1/2 Are Essential for RhoA/ROCK-Regulated Actomyosin Activity in Isolated Vascular Smooth Muscle Cells

**DOI:** 10.3390/cells9010132

**Published:** 2020-01-06

**Authors:** Lauren Porter, Rose-Marie Minaisah, Sultan Ahmed, Seema Ali, Rosemary Norton, Qiuping Zhang, Elisa Ferraro, Chris Molenaar, Mark Holt, Susan Cox, Samuel Fountain, Catherine Shanahan, Derek Warren

**Affiliations:** 1 British Heart Foundation Centre of Research Excellence, Cardiovascular Division, King’s College London, London SE5 9NU, UK; 2 School of Pharmacy, University of East Anglia, Norwich Research Park, Norwich NR4 7TJ, UK; 3 School of Biological Sciences, University of East Anglia, Norwich Research Park, Norwich NR4 7TJ, UK; 4 Randall Division of Cell and Molecular Biophysics, New Hunt’s House, King’s College London, London SE1 1YR, UK

**Keywords:** LINC complex, RhoA, actomyosin

## Abstract

Vascular smooth muscle cells (VSMCs) are the predominant cell type in the blood vessel wall. Changes in VSMC actomyosin activity and morphology are prevalent in cardiovascular disease. The actin cytoskeleton actively defines cellular shape and the LInker of Nucleoskeleton and Cytoskeleton (LINC) complex, comprised of nesprin and the Sad1p, UNC-84 (SUN)-domain family members SUN1/2, has emerged as a key regulator of actin cytoskeletal organisation. Although SUN1 and SUN2 function is partially redundant, they possess specific functions and LINC complex composition is tailored for cell-type-specific functions. We investigated the importance of SUN1 and SUN2 in regulating actomyosin activity and cell morphology in VSMCs. We demonstrate that siRNA-mediated depletion of either SUN1 or SUN2 altered VSMC spreading and impaired actomyosin activity and RhoA activity. Importantly, these findings were recapitulated using aortic VSMCs isolated from wild-type and SUN2 knockout (SUN2 KO) mice. Inhibition of actomyosin activity, using the rho-associated, coiled-coil-containing protein kinase1/2 (ROCK1/2) inhibitor Y27632 or blebbistatin, reduced SUN2 mobility in the nuclear envelope and decreased the association between SUN2 and lamin A, confirming that SUN2 dynamics and interactions are influenced by actomyosin activity. We propose that the LINC complex exists in a mechanical feedback circuit with RhoA to regulate VSMC actomyosin activity and morphology.

## 1. Introduction

Vascular smooth muscle cells (VSMCs) line the blood vessel wall and their function is regulated by both soluble and insoluble mechanical cues, including blood flow-derived stretch and matrix stiffness [1,2,3]. VSMCs normally exist in a quiescent, contractile phenotype and actomyosin activity drives VSMC contraction and regulates vascular tone and vessel compliance [4]. However, VSMCs are not terminally differentiated and changes in their mechanical environment promote VSMC dedifferentiation to a proliferative, synthetic phenotype [1,5]. Cell morphology is critical for normal tissue function and changes in VSMC morphology are observed during phenotypic modulation associated with development and disease [1]. The cytoskeleton actively shapes the cell via actomyosin generated force transmission between cell–matrix adhesions that span the plasma membrane and the extracellular matrix (ECM) [6]. Cell–matrix adhesions also serve as signalling hubs that sense and initiate cellular response to changes in the mechanical environment [7].

Analogous to cell–matrix adhesions, the LInker of Nucleoskeleton and Cytoskeleton (LINC) complex, spans the nuclear envelope (NE) and mechanically couples filamentous actin (F-actin) to the nuclear interior [8,9]. This complex is comprised of giant nesprin-1/2 isoforms and Sad1 and UNC84 domain containing proteins 1/2 SUN1/2 [8,9]. Nesprin-1/2 giant isoforms reside on the outer nuclear membrane (ONM) and bind F-actin via a pair of *N*-terminal Calponin homology (CH) domains [10]. The nesprin-1/2 giants also indirectly associate with F-actin, via interactions between nesprin spectrin repeats and F-actin binding proteins including the formin, formin homology 2 domain containing 1 (FHOD1), and the actin bundling protein, fascin [11,12]. LINC complex stability is maintained via interactions between the nesprin C-terminal Klarsicht, Anc-1, Syne-1 homology (KASH) domain, and the SUN domain of SUN1/2 in the perinuclear space [8,9]. SUN1/2 span the inner nuclear membrane (INM) and interact directly with lamin A/C, physically coupling the actin cytoskeleton to the nuclear lamina [13]. Importantly, the LINC complex transmits cytoskeletal-derived biophysical signals to the nuclear interior and several studies have shown that lamins A/C are regulated by actomyosin activity and cytoskeletal tension [14,15,16].

The LINC complex has emerged as a regulator of cytoskeletal organisation and SUN2 is essential for the mechanical integrity of intercellular adhesions suggesting that mechanical feedback exists between the NE and cell–cell contacts [17]. Feedback also exists between the NE and matrix adhesions and disruption of lamins A/C function or overexpression of the dominant negative nesprin KASH-domain triggers focal adhesion reorganisation and changes in cell motility [18,19,20]. The nature of this feedback remains unknown, however, lamin A/C and SUN2 have recently been demonstrated to regulate Rac1 and RhoA activity, respectively [20,21]. Although in some contexts LINC complex components are partially redundant, several lines of data suggest that the LINC complex is tailored for cell-type-specific functions. For example, nesprin isoform switching is observed during muscle development [22,23]. Data also suggest that SUN1 and SUN2 perform specific roles in tailoring LINC complex function; SUN2 has a specific role in nuclear positioning in polarising fibroblasts, SUN1 has a specific role in nuclear pore assembly and distribution and SUN1 and SUN2 have opposing roles in RhoA regulation in HELA cells [21,24,25]. Recent findings have also suggested that SUN1 and SUN2 containing LINC complexes are associated with different cytoskeletal systems. Studies using fibroblast cells have shown that SUN1/nesprin-1 LINC complexes associate with microtubules, whereas SUN2/nesprin-2 LINC complexes associate with the perinuclear actin cap [24,26].

Whether the LINC complex contributes to VSMC actomyosin activity remains unknown. Therefore, we sought to define the roles of SUN1 and SUN2 in VSMC nuclear envelope/cytoskeletal coupling and actomyosin-mediated force generation. We demonstrate that RhoA/rho-associated, coiled-coil-containing protein kinase (ROCK)-signalling and SUN1/2 form a biophysical signalling circuit that regulates VSMC spreading and actomyosin activity.

## 2. Materials and Methods

### 2.1. Animals

Experiments were carried out according to the Animals (Scientific Procedures) Act 1986 and were ethically approved by the King’s Animal Care Research Ethics Committee. Wild-type (WT) and SUN2 knockout (SUN2 KO) B6;12956-SUN2tm1MHan/J animals (male, 2–3 months) have been characterised previously and were purchased from The Jackson Laboratory (Sacramento, CA, USA) [27]. 

### 2.2. Cell Culture

Human aortic VSMCs (passage 7–11) and mouse aortic VSMCs (passage 4–10) were cultured as described previously [28,29]. The stiffness of the aortic wall has been measured previously to be 10–20 kPa, therefore, VSMCs were grown on 12 kPa throughout this study unless otherwise stated [30]. Dharmacon SUN1 and SUN2 smart pool siRNA were used in this study. Transfection of siRNA was performed using HiPerFect (Qiagen, Hilden, Germany), as per manufacturer’s instructions. ROCK and myosin II inhibition was achieved by treating cells with 5 µM Y27632 or 40 µM blebbistatin (Sigma-Aldrich, St Louis, MO, USA), for 30 min unless otherwise stated. 

### 2.3. Polyacrylamide Hydrogel Preparation and Traction Force Microscopy (TFM)

Hydrogels were prepared as described previously [31,32]. A JPK Nanowizard-3 atomic force microscope (Brucker Nano GmbH, Berlin, Germany) was used to confirm hydrogel stiffness as described previously [31]. VSMCs were seeded onto 12 kPa hydrogels containing 0.5 µM red fluorescent (580/605) FluoSpheres (Invitrogen, Carlsbad, CA, USA). Imaging was performed using a Nikon Eclipse Ti-E live cell imaging system to capture 20× magnification images which were captured every 60 s for 30 min after addition of the ROCK inhibitor Y27632 or the myosin II inhibitor, blebbistatin. To determine total traction stress, images were captured before and after lysing cells in 0.5% Tx-100 (Sigma-Aldrich, St Louis, MO, USA). Drift was corrected using the ImageJ StackReg plugin and traction stress was calculated using the ImageJ plugin described previously to measure FluoSphere displacement [33]. The bead displacement was measured using the first and last image of the movie sequence. To determine integrated (cell total), maximum, and cell average traction stresses, the cell region was segmented by overlaying the cell image with the traction map, highlighting the cell traction region with a region of interest (ROI) and extracting the traction stresses in each pixel by using the save XY coordinate function in ImageJ. 

### 2.4. Confocal Microscopy and Analysis, Western Blotting, qPCR Analysis, and RhoA Activity Assays

Antibodies used for western blotting (WB), IF; α-actin, β-actin (A5316) (Sigma), calponin, SUN2 (Abcam: ab124916), SUN1 (Abcam: ab103021), and nesprin-2 CH3 (characterised previously [10]). Secondary antibodies for WB were horseradish peroxidase-conjugated anti mouse (NA931) or anti-rabbit (NA94V) antibodies from GE Healthcare. Enhanced chemiluminescent kit (RPN2132, GE Healthcare) was used for detection according to manufacturer’s instructions. Invitrogen anti-mouse Alexa Fluor 568 (A11031) and anti-rabbit Alexa Fluor 488 (A11034) were used as immune fluorescence secondary antibodies. For cell morphology analysis, VSMCs were cultured on hydrogels, fixed in 4% paraformaldehyde and processed as described previously. Images were captured at 20× magnification using a Leica SP5 laser scanning confocal microscope (Leica Microsystems GmbH, Wetzlar, Germany). Cells were counterstained with SUN1 or SUN2 antibodies and only cells lacking staining in the knockdown conditions were analysed. Cell area and circularity (1 is a perfect circle and 0 a straight line) was measured using the find object function in Volocity analysis software (Quorum Technologies Inc, Puslinch, ON, Canada). To visualise the perinuclear actin cap, VSMCs were fixed and processed as described above. Images were captured on an ×20 objective and image stacks were taken at 0.4 µm intervals. Cell and nuclear volume were measured using the Volumest ImageJ plugin [34]. Western blotting (WB) and qPCR were performed as described previously, using Qiagen primer pairs for ACTA2, MYH9, and ACTB [28]. qPCR target mRNA was normalised to GAPDH mRNA levels. RhoA activity was measured using RhoA GLISA (GTP-bound) and ELISA (Total) kits as per manufacturer’s instructions (Cytoskeleton Inc., Denver, CO, USA).

### 2.5. Fluorescence Recovery after Photo Bleaching (FRAP) and Fluorescence Resonance Energy Transfer (FRET) Analysis

Red fluorescent protein (RFP)-lamin A and Green fluorescent protein (GFP)-SUN2 (a kind gift from Prof. Howard Worman, Columbia University) and GFP-SUN1 (a kind gift from Dr. Sue Shackleton, University of Leicester) were transfected into VSMCs using Fugene6 transfection reagent. FRAP was performed using the FRAP application module on a Leica SP5 laser scanning microscope (Leica Microsystems GmbH, Wetzlar, Germany). Images were captured every 380 ms using a ×63 objective—10 pre-bleach and 200 post-bleach images were captured. Regions of interest (ROI) were bleached at full laser power and post-bleach images were captured every 380 ms. FRAP analysis was performed to calculate mobile fraction and T1/2 using the ImageJ FRAP profiler plugin (The Hardin laboratory website: http://worms.zoology.wisc.edu/research/4d/4d.html#frap). Acceptor photo-bleaching FRET analysis was performed on GFP-SUN2/RFP-lamin A expressing VSMCs grown on 12 kPa hydrogels 48 h post-transfection using the Leica Acceptor Bleaching Wizard. Cells were fixed in 4% paraformaldehyde and washed in Phosphate Buffered Saline. Cells with comparable levels of GFP/RFP expression were selected for FRET analysis. The RFP signal was bleached by repeatedly illuminating the cell at 100% laser power. Images were obtained before (pre-bleach) and after (post-bleach) acceptor photo-bleaching. The nuclear lamina was selected using an ROI and FRET efficiency was calculated using the equation: FRETeff = Donorpost-bleach − Donorpre-bleach/Donorpost-bleach, where Donorpre-bleach corresponds to the fluorescence intensity of the donor (GFP-SUN2) before acceptor photo-bleaching and Donorpost-bleach corresponds to the fluorescence intensity of the donor after acceptor photo-bleaching.

### 2.6. Statistical Analysis

Results are presented as mean ± Standard error of the mean. For comparison of siRNA knockdown groups and control and inhibitor-treated groups. Box and whisker plots represent the following: Boxes represent the first and third quartile of the data. The band inside represents the median of the data. Whiskers represent the minimum and maximum values of all the data. Paired Student’s t-tests or one-way ANOVA with Bonferroni post hoc test was performed on the data sets.

## 3. Results

### 3.1. Disruption of SUN1 or SUN2 Alters Perinuclear Actin Cap Organisation in Synthetic VSMCs

Recent studies have shown that SUN1 and SUN2 possess independent functions, therefore, we set out to confirm that VSMCs possess SUN1 and SUN2. qPCR analysis and western blot (WB) confirmed the expression of both SUN1 and SUN2 in isolated human VSMCs; however, SUN2 was more abundantly expressed than SUN1 (Supplementary Figure S1A,B). We next sought to confirm the validity of our antibodies, using human dermal fibroblast and human bone osteosarcoma epithelial (U2OS) cells. WB analysis revealed that SUN1 and SUN2 are both abundant in these cell types, confirming that SUN1 was expressed at lower abundance than SUN2 in isolated VSMCs (Supplementary Figure S1C). The LINC complex associates with actin cables that overlay the nucleus (termed actin caps), and we next confirmed that VSMCs possess actin caps [35]. Confocal immunofluorescence microscopy, imaging the apical, middle, and basal positions of VSMCs, confirmed that VSMCs possess actin cables overlaying their nuclei (Supplementary Figure S2).

**Figure 1:**
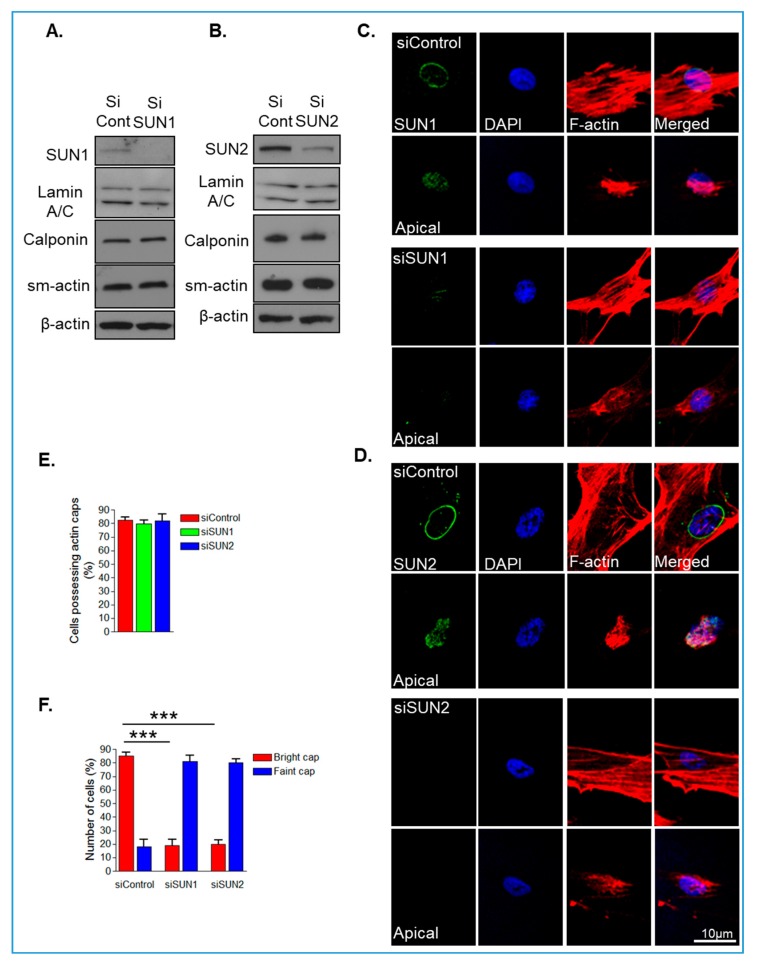
SUN1 or SUN2 depletion induces actin cap reorganisation. Western blotting (WB) of (**A**) SUN1- and (**B**) SUN2-depleted vascular smooth muscle cells (VSMC) lysates. Confocal immunofluorescence analysis imaging the apical and middle sections of F-actin (red), SUN1 or SUN2 (green), and DAPI (blue) stained (**C**) control and SUN1-depleted and (**D**) control and SUN2-depleted VSMCs grown on 12 kPa hydrogels. Graphs show (**E**) number of VSMC possessing actin caps and (**F**) the number of VSMCs possessing bright or faint actin cap staining displayed by control, SUN1- and SUN2-depleted VSMCs grown on 12 kPa hydrogels. Graphs represent the combined data from three independent experiments analysing >20 cells (*** *p* < 0.0001).

The above data demonstrate that SUN2 is more abundant in isolated human VSMCs than SUN1. Recent studies have shown that SUN1 and SUN2 possess specific functions at the NE, so we next investigated whether disruption of SUN1 or SUN2 individually influenced organisation of the actin cap, by employing an siRNA-mediated depletion strategy. WB confirmed efficient depletion of SUN1 and SUN2 after siRNA treatment in VSMCs (Figure 1A,B). We next assessed the impact of SUN1 or SUN2 depletion on levels of lamins A/C and the actomyosin contractile apparatus. qPCR analysis revealed that SUN1 and SUN2 depletion did not alter mRNA levels of smooth muscle actin (sm-actin), β-actin, or non-muscle-myosin II (NM-myosin II) (Supplementary Figure S3). WB confirmed that levels of lamins A/C and the actomyosin contractile proteins calponin, sm-actin, and β-actin remained unaltered in SUN1- and SUN2-depleted VSMCs (Figure 1A,B). 

Next, we examined the impact of SUN1 and SUN2 depletion on organisation of the LINC complex and perinuclear actin cap. IF microscopy revealed that SUN1- and SUN2-depleted VSMCs retained nesprin-2 staining at the NE (Supplementary Figure S4), suggesting that the LINC complex remains intact. Confocal IF, imaging the middle and apical planes of VSMCs, revealed that SUN1- and SUN2-depleted VSMCs possessed actin caps and there was no change in alignment of global actin and actin caps (Figure 1C–E and Supplementary Figure S5). However, closer examination revealed that control VSMCs displayed strong actin cap staining whereas SUN1- and SUN2-depleted VMSCs displayed faint actin cap staining, suggesting that the actin cap is reorganised in SUN1- and SUN2-depleted VSMCs (Figure 1C,D,F and Supplementary Figure S5). 

### 3.2. SUN1 and SUN2 Influence VSMC Spreading

**Figure 2:**
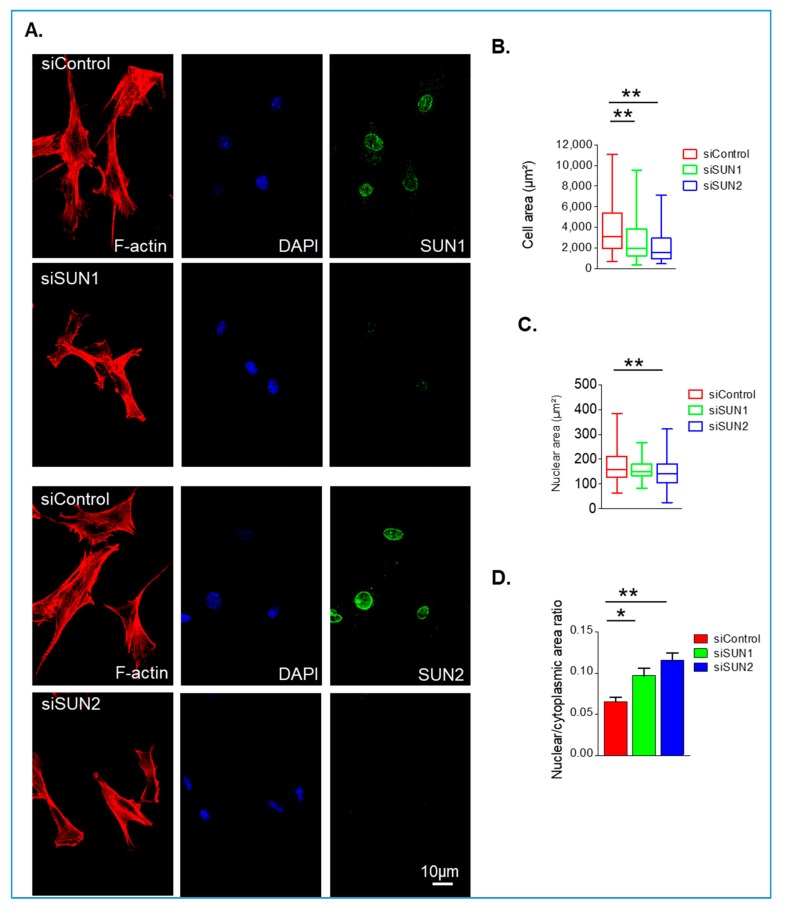
SUN1 and SUN2 influence isolated VSMCs spreading. (**A**) Representative confocal immunofluorescence microscopy images of rhodamine phalloidin (red), SUN1 or SUN2 (green), and DAPI (blue) stained VSMCs grown on 12 kPa hydrogels. Graphs show IF analysis of (**B**) cell area, (**C**) nuclear area, and (**D**) nuclear area:cytoplasmic area ratio of control, SUN1- and SUN2-depleted VSMCs. Graphs represent combined data of three independent experiments analysing >300 cells per group (* *p* < 0.05 and ** *p* < 0.01).

The above data show that the perinuclear actin cap is reorganised in VSMCs depleted of either SUN1 or SUN2. Next, we investigated whether SUN1 and SUN2 influence VSMC morphology and show that depletion of either reduced the cellular area of VSMCs (Figure 2A,B). Analysis of 3D confocal stacks revealed that although cellular area had reduced, cell volume remained unchanged in SUN1- and SUN2-depleted VSMCs compared to controls (Supplementary Figure S6A,B). In addition, SUN2-depleted cells also displayed a reduction in nuclear area (Figure 2A,C), however, nuclear volume remained unaltered (Supplementary Figure S6A,C). Analysis of the nuclear/cytoplasmic ratio revealed that SUN1- and SUN2-depleted VSMCs displayed an increased ratio of nuclear/cytoplasmic area (Figure 2D), suggesting that SUN1 and SUN2 have a greater influence on spreading of the cytoplasm than on the nucleus. Importantly, nuclear/cytoplasmic volume remained unchanged, confirming that overall scaling between the cytoplasm and nucleus remained constant (Supplementary Figure S6A,D).

**Figure 3:**
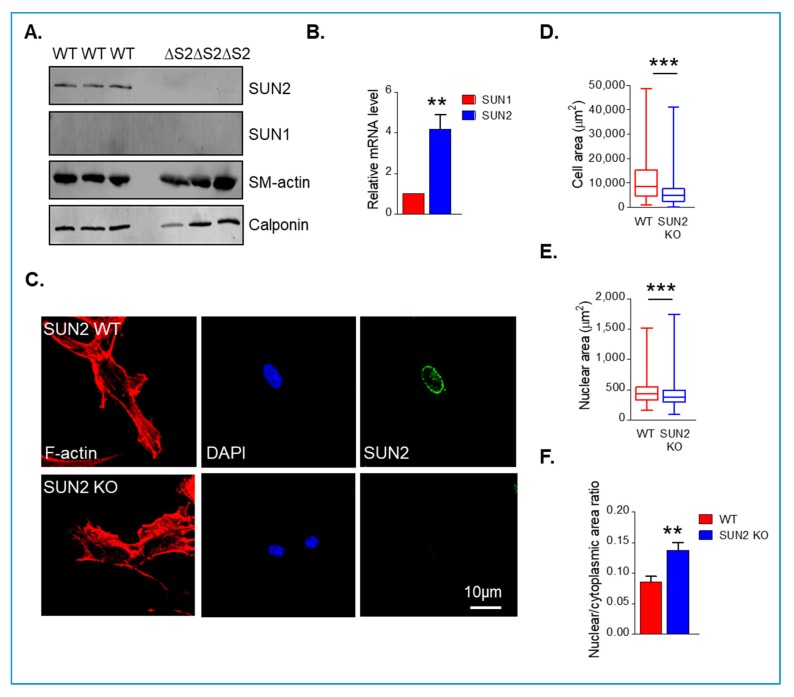
SUN2 KO VSMCs display reduced spreading. (**A**) WB of wild-type (WT) and SUN2 KO aortic samples. Each lane corresponds to an independent aortic sample isolated from different WT and SUN2 KO mice. (**B**) qPCR analysis of SUN1 and SUN2 mRNA expression in WT aortae. (**C**) Representative images of isolated WT and SUN2 KO mouse aortic VSMCs stained for F-actin (red), SUN2 (green), and DAPI (blue). Graphs show (**D**) cell area, (**E**) nuclear area, and (**F**) nuclear area:cell area ratio of WT and SUN2 KO VSMCs. Graphs represent the combined data of three independent experiments analysing >200 cells per group (** *p* < 0.01 and *** *p* < 0.0001).

**Figure 4:**
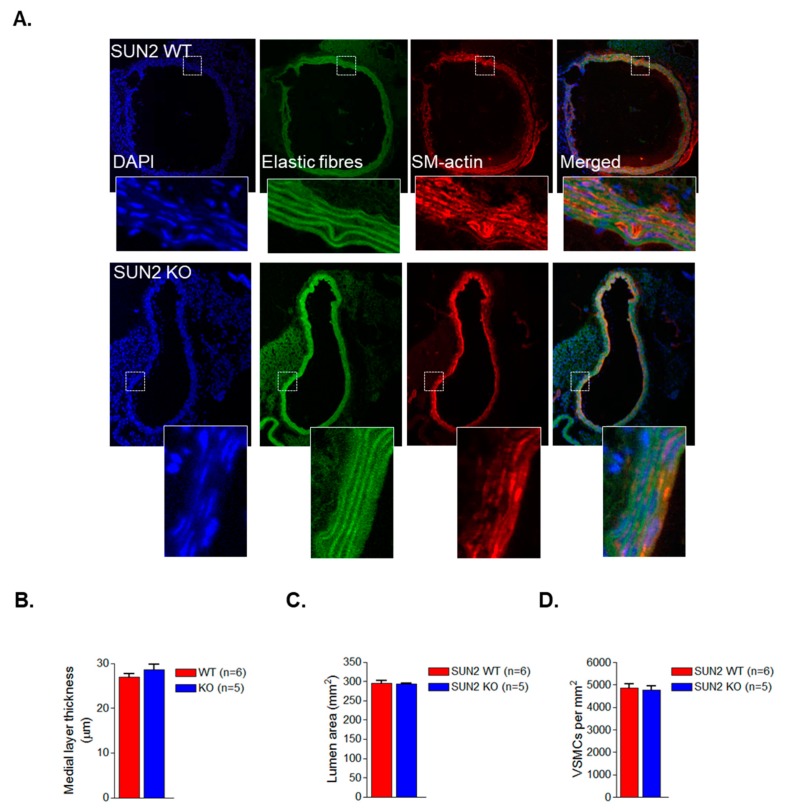
SUN2 KO aortae display normal VSMC organisation. (**A**) Representative IHC images of WT and SUN2 KO aortae stained with DAPI (blue), smooth muscle (sm)-actin (red), and the elastic fibre auto-fluorescence (green). Graphs show (**B**) medial layer thickness, (**C**) lumen area, and (**D**) VSMC number in WT and SUN2 KO aortae.

The above data show that SUN1 and SUN2 influence VSMC spreading. To further confirm these findings, we utilised the global SUN2 KO mouse model described previously [27]. WB revealed that SUN2 was present in wild-type aortae however, SUN1 was not detected (Figure 3A). To further confirm SUN2 was more abundant in mouse aortae, we examined the level of mRNA present. qPCR analysis confirmed that SUN2 was more abundant than SUN1 in mouse aortae (Figure 3B). Furthermore, WB confirmed the absence of SUN2 in aortae isolated from SUN2 KO mice (Figure 3A). WB also showed that SUN2 KO aortae possessed similar levels of the contractile proteins sm-actin and calponin (Figure 3A). To observe whether VSMC spreading was altered, we isolated VSMCs from aortae of SUN2 KO mice. Similar to SUN2 depleted VSMCs, analysis confirmed that SUN2 KO VSMCs displayed a reduction in cellular and nuclear area (Figure 3C–E). Similar to the SUN1- and SUN2-depleted VSMCs, SUN2 KO VSMCs displayed an increased nuclear/cytoplasmic area ratio (Figure 3F). Next, we postulated that if cytoplasmic/nuclear scaling remained unaltered there would no change in VSMC numbers in SUN2 KO aortae. To investigate this possibility, we performed immunohistochemistry analysis of SUN2 WT and SUN2 KO aortae. Analysis revealed that SUN2 KO aortae possessed similar medial layer thickness, lumen area, and VSMC number/area as SUN2 WT aortae (Figure 4A–D).

### 3.3. SUN1/2 Influence RhoA and Actomyosin Activity in Synthetic VSMCs

The above data demonstrate that SUN1 and SUN2 influence cell spreading. Actomyosin generated force is a major contributor to cell spreading, so we next investigated whether actomyosin regulated VSMC spreading. To define the pathways contributing to isolated VSMC actomyosin activity, we performed traction force microscopy (TFM) using detergent (total traction stress (TS)), Y27632 (ROCK-mediated TS), and blebbistatin (myosin II-mediated TS) to relax VSMC traction stresses. TFM confirmed that integrated, maximal, and cell average traction stresses for blebbistatin- and Y27632-treated VSMCs were similar to those measured for total VSMC traction stress (Supplementary Figure S7A–D). These data confirm that isolated VSMC traction stresses are predominantly regulated by a ROCK/myosin II dependent pathway. To confirm whether actomyosin activity influences VSMC spreading, we analysed the area of blebbistatin- and Y27632-treated VSMCs and show that actomyosin inhibition resulted in a reduction in cellular area compared to control VSMCs (Supplementary Figure S8A,B). Nuclear area remained unaltered and the nuclear area/cytoplasmic area ratio was increased compared to control VSMCs (Supplementary Figure S8A,C,D). Further characterisation revealed that that SUN1 and SUN2 remained localised at the NE in blebbistatin- and Y27632-treated VSMCs (Supplementary Figure S9A). qPCR analysis confirmed that levels of sm-actin, NM-myosin II, and β-actin remained unaltered by blebbistatin and Y27632 treatments (Supplementary Figure S9B). Furthermore, WB confirmed that SUN1 and SUN2, lamins A/C and the contractile protein sm-actin remained unaltered by blebbistatin and Y27632 treatments (Supplementary Figure S9C).

**Figure 5:**
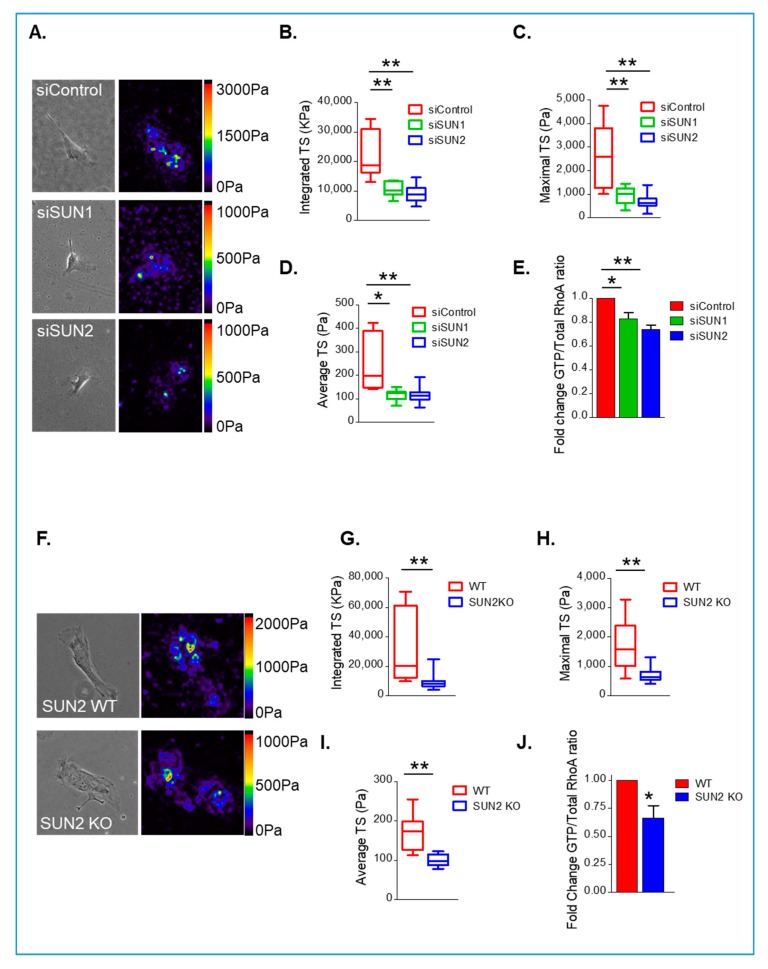
SUN1 and SUN2 influence actomyosin and RhoA activity. (**A**) Representative traction stress heat map of Y27632-treated control, SUN1-, and SUN2-depleted VSMCs grown on 12 kPa hydrogels. Graphs show (**B**), integrated-TS, (**C**), maximal-TS, and (**D**), cell average-TS of control (*n* = 18), SUN1-depleted (*n* = 15), and SUN2-depleted (*n* = 17) isolated VSMCs (* *p* < 0.05 and ** *p* < 0.01). (**E**) Graph shows RhoA activity (GTP-RhoA:total RhoA ratio) in control, SUN1-, and SUN2-depleted VSMCs and represents the combined data from three independent experiments repeated in triplicate (* *p* < 0.05 and ** *p* < 0.01). (**F**) Representative traction stress heat maps of WT and SUN2 KO VSMCs grown on 12 kPa hydrogels. Graphs show (**G**), integrated-TS, (**H**), maximum-TS, and (**I**), cell average-TS of WT (*n* = 10) and SUN2 KO (*n* = 11) mouse VSMCs (** *p* < 0.01). (**J**) Graph shows RhoA activity (GTP-RhoA:total RhoA ratio) in WT and SUN2 KO mouse VSMCs. Graph represents combined data from three independent experiments repeated in triplicate (* *p* < 0.05).

The above data demonstrate that actomyosin activity influences VSMC spreading, therefore, we next predicted that SUN1 and SUN2 influence actomyosin activity in VSMCs. To examine this possibility, we performed TFM on SUN1- and SUN2-disrupted VSMCs. TFM analysis revealed that integrated, maximal, and cell average traction stresses were attenuated in SUN1-depleted, SUN2-depleted (Figure 5A–D), and SUN2 KO (Figure 5F–I) VSMCs, compared to their control and WT counterparts, respectively. Importantly, SUN1-depleted, SUN2-depleted, and SUN2 KO VSMCs displayed impaired RhoA activity compared to their control and WT counterparts (Figure 5E,J), suggesting that both SUN1 and SUN2 influence RhoA and actomyosin activity in isolated VSMCs.

### 3.4. Actomyosin Activity Regulates SUN2/Lamin A Association in Isolated VSMCs

The above data confirm that SUN1 and SUN2 influence actomyosin activity in proliferative VSMCs. Next, we postulated that actomyosin and SUN1/2 form a bidirectional signalling loop and that actomyosin activity would influence SUN1/2 dynamics at the NE. To examine this possibility, we transfected GFP-tagged SUN1 and SUN2 constructs into VSMCs. We found that 24 h after transfection, both GFP-tagged SUN1 and SUN2 localised to the NE and the endoplasmic reticulum (Supplementary Figure S10A). GFP-SUN1 localisation remained in both the NE and ER 48 h after transfection; however, GFP-SUN2 was predominantly localised at the NE at this time point (Supplementary Figure S10A). Analysis of nuclear morphology of GFP-SUN2-transfected VSMCs revealed that nuclear area decreased 24 h after transfection compared to controls, suggesting that GFP-SUN2 overexpression was disrupting NE organisation. However, nuclear area returned to that of the control VSMCs 48 h after transfection (Supplementary Figure S10B). Consistent with our previous findings, GFP-SUN2 remained localised at the NE during both blebbistatin and Y27632 treatments (Supplementary Figure S10C). This data suggests that SUN1/2 expression level influences VSMC nuclear morphology, therefore, subsequent experiments were performed using the GFP-tagged SUN2 48 h after transfection.

**Figure 6:**
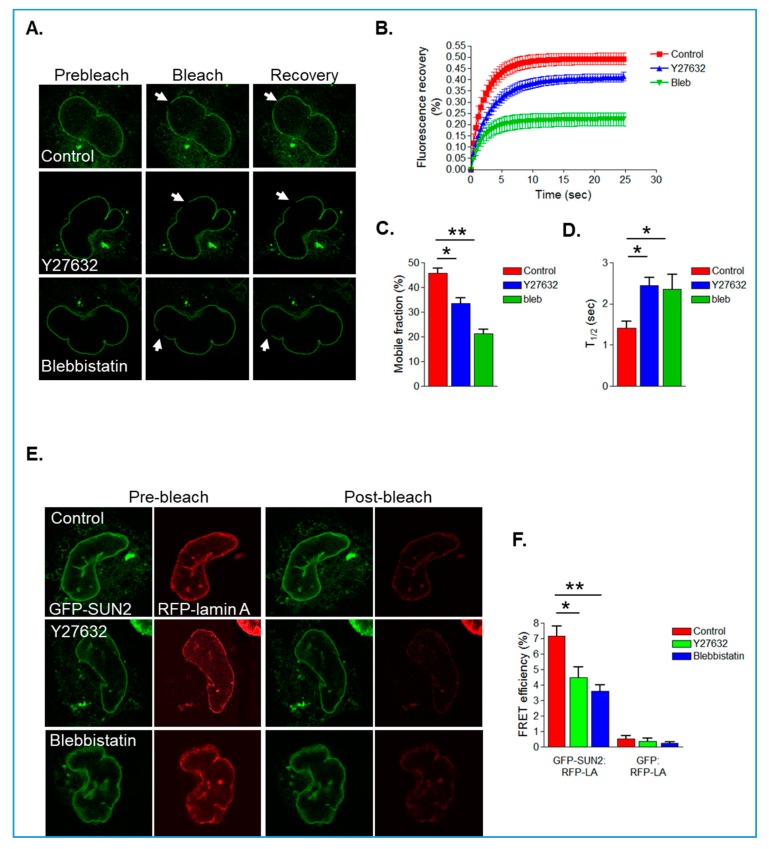
Actomyosin activity increases SUN2 mobility and the interaction between SUN2 and lamin A. (**A**) Representative prebleach, bleach, and recovery images of control, Y27632- and blebbistatin-treated GFP-SUN2 expressing VSMCs grown on 12 kPa hydrogels (arrow marks the bleached region). Graphs show (**B**) fluorescence recovery profile, (**C**) mobile fraction, and (**D**) T1/2 of GFP-SUN2 recovery in control (*n* = 21), Y27632 (*n* = 18)-, and blebbistatin (*n* = 22)-treated VSMCs (* *p* < 0.05 and ** *p* < 0.01). (**E**) Representative prebleach and post-bleach images of control, Y27632-, and blebbistatin-treated VSMCs expressing GFP-SUN2 and RFP-lamin A grown on 12 kPa hydrogels. (**F**) Graph shows GFP-SUN2/RFP-lamin A and GFP/RFP-lamin A FRET efficiency of control (*n* = 22), Y27632 (*n* = 19)-, and blebbistatin (*n* = 24)-treated VSMCs (* *p* < 0.05 and ** *p* < 0.01).

To analyse the impact of actomyosin on GFP-SUN2 mobility within the NE, we performed fluorescence recovery after photo bleaching (FRAP) analysis. Both blebbistatin and Y27632 treatment resulted in a reduction in GFP-SUN2 mobile fraction and an increase in T1/2, suggesting that actomyosin activity promotes SUN2 mobility in the NE (Figure 6A–D). Previous studies have shown that lamin A interacts with SUN2 and regulates the mobility of SUN2 in the INM [29]. Therefore, we next examined whether actomyosin activity influences the association between SUN2 and lamin A by performing acceptor photo-bleaching FRET analysis on VSMCs co-expressing GFP-SUN2 and RFP-lamin A. Similar to previous studies, GFP-SUN2 and RFP-lamin A had a FRET efficiency of around 7% in control VSMCs (Figure 6E,F). However, blebbistatin- and Y27632-treated VSMCs displayed a reduction in FRET efficiency between GFP-SUN2 and RFP-lamin A (Figure 6E,F).

## 4. Discussion

Our knowledge of how the NE couples to the cytoskeleton has increased dramatically, and differences in LINC complex composition are observed between cell types. In this study, we investigated the individual roles of SUN1 and SUN2 in VSMC function. We found differences in SUN1 and SUN2 expression levels; SUN2 was more abundant than SUN1 is isolated VSMCs and SUN2 was highly expressed in aortae but SUN1 was not detected. These data suggest that SUN1 expression may be linked to VSMC phenotype. Within the aortae, VSMCs exist in a quiescent, contractile phenotype, in contrast, isolated VSMCs adopt a proliferative, synthetic phenotype [5]. Expression of genes involved in actomyosin contraction and proliferation are altered during VSMC phenotypic modulation and potentially SUN1 expression is regulated by this process. In support of this notion, the giant nesprin-1 isoform was originally identified in a screen for proteins highly expressed in contractile VSMCs, further suggesting the LINC complex is tailored for different VSMC phenotypes [36].

Disruption of SUN1 or SUN2 in isolated VSMCs resulted in decreased RhoA and actomyosin activity, suggesting that SUN1 and SUN2 both regulate RhoA and actomyosin activity. Previous studies have demonstrated that SUN-domain family members form trimers that bind three separate KASH domains [37], suggesting that SUN2 homotrimers influence RhoA and actomyosin activity in contractile VSMCs. In contrast, disruption of either SUN1 or SUN2 in isolated VSMCs resulted in similar reductions in RhoA and actomyosin activity, suggesting that SUN1 and SUN2 heterotrimers regulate RhoA and actomyosin activity in isolated VSMCs. Our findings are in contrast to a recent study using HELA cells that reported that SUN1 antagonises SUN2 function and inhibits RhoA activity [21]. This discrepancy further suggests that SUN1- and SUN2-containing LINC complexes may be tailored for cell-type-specific functions. In further support of this notion, previous studies have shown that LINC complex disruption in different cell types has different effects on actomyosin activity; nesprin disruption enhances actomyosin activity in skeletal muscle progenitor and endothelial cells. Lamin A disruption in skeletal muscle progenitor cells also enhanced actomyosin activity. In contrast, lamin A disruption reduced actomyosin activity in fibroblast cells [17,38,39]. Thus, our understanding of the balance between LINC complex function/composition and actomyosin activity is complex and remains to be fully defined in cell-specific contexts.

We also demonstrate that disruption of either SUN1 or SUN2 alone failed to displace nesprin-2 from the ONM in isolated VSMCs, suggesting that the LINC complex remains intact. Potentially, the loss of either SUN1 or SUN2 results in increased formation of homotrimers, which interact with the KASH domain and continue to anchor nesprin isoforms to the NE. Importantly, the potential formation of homotrimers in isolated VSMCs failed to compensate the RhoA and actomyosin activities. SUN1 was absent in aortic samples in which contractile VSMCs are the predominant cell type. This suggests that contractile VSMCs possess SUN2 homotrimers, whereas synthetic VSMCs possess both SUN1/2 heterotrimers and SUN2 homotrimers. As discussed above, potentially, VSMC phenotypic modulation results in altered expression of NE components and NE interacting proteins. In agreement with this notion, we show that SUN1 was expressed in isolated VSMCs but lacking in contractile VSMCs. However, VSMC phenotypic modulation also results in altered myosin expression. Contractile VSMCs possess both smooth muscle-myosin (SM-myosin) and non-muscle myosin II (NM-myosin) whereas proliferative VSMCs predominantly possess NM-myosin II [40]. Therefore, LINC complex composition is potentially tailored for different myosin isoforms. Previous studies have shown that NM-myosin generates less force than SM-myosin and that NM-myosin II contributes to tonic contraction whereas SM-myosin contributes to phasic contraction [40,41,42]. Potentially, interactions between SUN1:SUN2 and SUN2:SUN2 have different stabilities, allowing the LINC complex to resist changes in actomyosin activity associated with different myosin types; however, this possibility remains untested. Further investigation is now required to further define how the LINC complex is tailored to support changes in myosin activity in contractile and proliferative VSMC function.

### SUN2/RhoA Crosstalk

In this study, we investigated the relationship between SUN1/2 mechanical coupling and actomyosin activity. Our findings suggest that RhoA signalling and SUN1/2 form a bidirectional mechano-signalling circuit that regulates actomyosin activity. Intracellular and extracellular derived mechanical forces are sensed by focal adhesions (FAs) and induce conformational changes in proteins such as filamin-A that promote rapid activation of RhoA to stimulate actomyosin activity and increase F-actin stiffness [43,44,45,46]. These biophysical signals are sensed by the LINC complex and we show that ROCK-induced actomyosin activity increases both SUN2 mobility and the association between SUN2 and lamin A at the INM. This suggests that actomyosin-generated forces tug on SUN2, resulting in an increased coupling between SUN2 and lamin A. These data are consistent with previous studies performed on isolated nuclei that show that direct transmission of mechanical force across nesprin-1 on the ONM increase recruitment of lamin A to the INM [16].

Importantly, our findings demonstrate that disruption of SUN1 or SUN2 impaired RhoA activity and reduced traction forces generated by VSMCs. These data suggest that the LINC complex is a bidirectional signalling hub and that SUN1/2 exist in a biophysical feedback circuit with RhoA to regulate VSMC actomyosin activity. We propose that these proteins form a biophysical feedback circuit to synchronise changes in extracellular and intracellular environments. While the nature of this feedback signal remains unknown, we show that SUN2 mobility and SUN2 association with lamin A is increased by actomyosin activity, suggesting that the LINC complex is subjected to mechanical load. In agreement with this notion, the LINC complex has recently been shown to be subjected to mechanical tension [47]. Other studies have demonstrated that changes in actomyosin activity regulates lamin A stability; lamin A is phosphorylated and degraded in conditions of low actomyosin activity, whereas high actomyosin activity increases lamin A levels [14,15]. Potentially, the increased association between lamin A and the INM stimulated by increased actomyosin activity protects lamin A from phosphorylation and ultimately leads to increased lamin A levels.

We propose that enhanced association between lamin A and SUN2 will increase LINC complex tension. An intriguing possibility is that LINC complex tension influences the conformation of mechano-sensors at FAs. Indeed, disruption of lamins A/C or expression of the nesprin KASH-domain alters focal adhesion organisation, and both lamins A/C and SUN2 have recently been reported to influence Rac1 and RhoA activity, respectively [20,21]. This suggests that the Rho-family of small GTPases are key players in this feedback pathway, however, further experimentation is now required to define the nature of the feedback signal and whether biophysical feedback signals regulate VSMC-matrix mechano-sensors.

## Supplementary Materials

The following are available online at https://www.mdpi.com/2073-4409/9/1/132/s1. Figure S1: Characterisation of SUN1 and SUN2 expression in isolated VSMCs; Figure S2: Characterisation of SUN1, SUN2 and the actin cap in isolated VSMCs; Figure S3: QPCR analysis of sm-actin, myosin II and β-actin mRNA level in control, SUN1 and SUN2 depleted VSMCs; Figure S4: Nesprin-2 remains localised to the NE in SUN1 and SUN2 depleted VSMCs; Figure S5: SUN1 or SUN2 depletion does not alter F-actin alignment in isolated VSMCs; Figure S6: SUN1 and SUN2 do not influence isolated VSMC volume; Figure S7: Traction stress is regulated by a ROCK/myosin II pathway in isolated VSMCs; Figure S8: VSMC area is regulated by a ROCK/myosin II pathway; Figure S9: Actomyosin activity does not affect SUN1/2 NE localisation; Figure S10: SUN2 NE localisation is restored 48 hours after transfection.
