# Supplementary material for: SUN1/2 Are Essential for RhoA/ROCK-Regulated Actomyosin Activity in Isolated Vascular Smooth Muscle Cells
Source: Cells. 2020 Jan 6;9(1):132. doi: 10.3390/cells9010132 (PMC7017107; doi:10.3390/cells9010132)
Supplement: Supplementary file 1 [file cells-09-00132-s001.pdf]

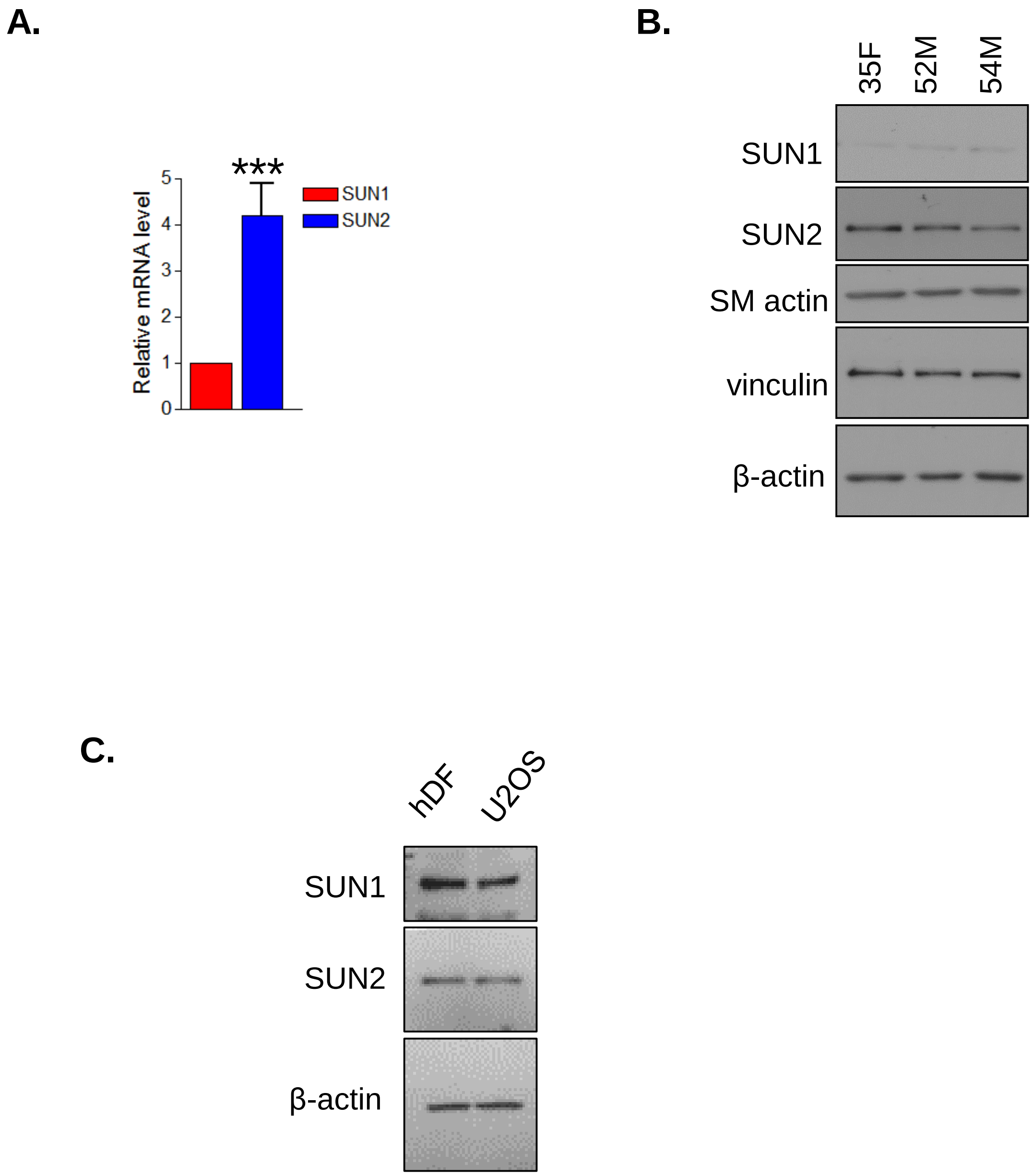

Supplementary Figure S1. Characterisation of SUN1 and SUN2 expression in isolated VSMCs. (A) QPCR analysis of SUN1 and SUN2 mRNA level in isolated 35F donor VSMCs. Graph consists of 3-independent experiments repeated in triplicate. (***p = <0.0001). (B) WB of 3 different VSMC isolates from 3 different donors (35F, 52M and 54M) for expression of SUN1, SUN2 and the contractile proteins α-actin and β-actin. (C) WB of SUN1 and SUN2 in human dermal fibroblast (hDF) and U2OS cells.

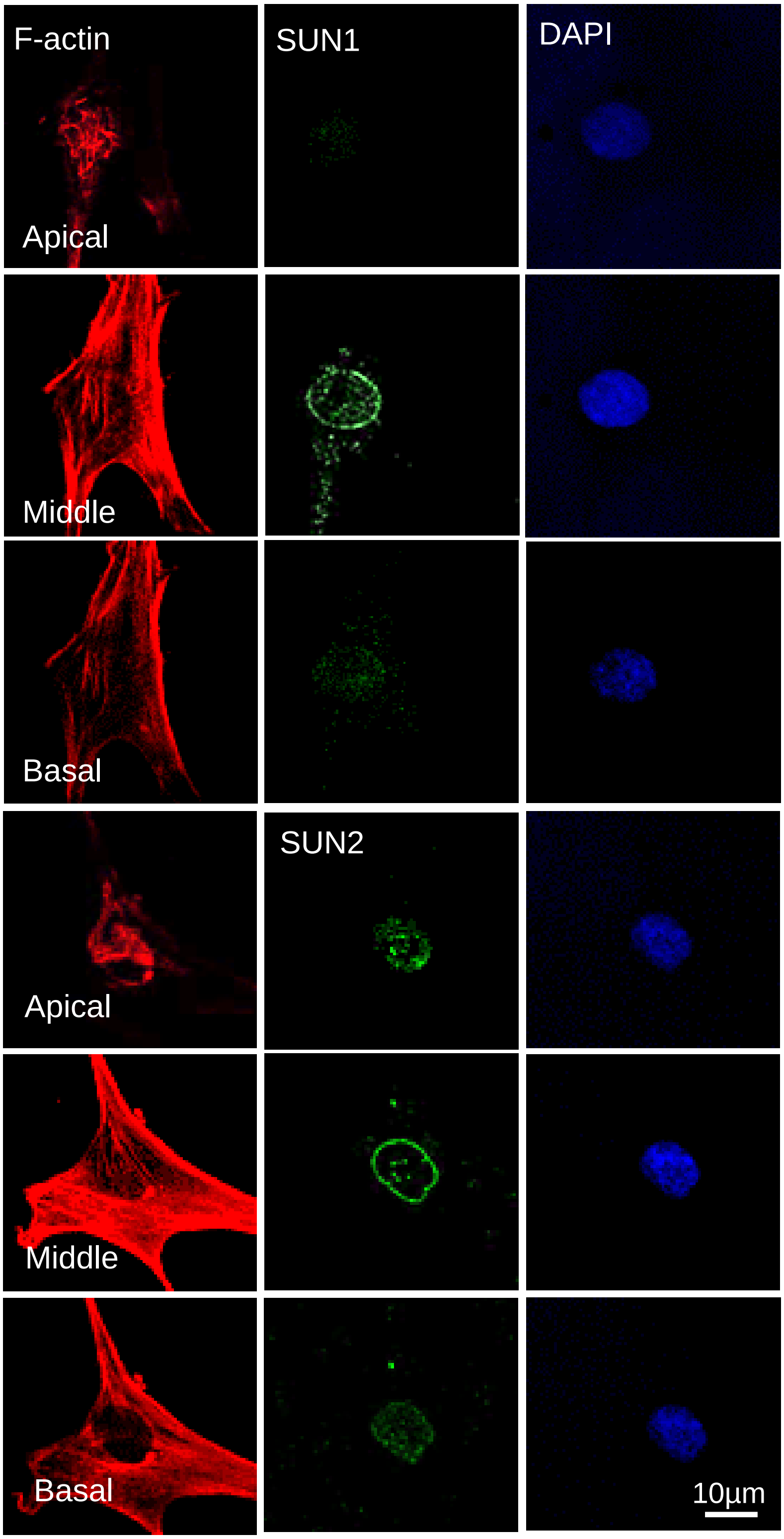

Supplementary Figure S2. Characterisation of SUN1, SUN2 and the actin cap in isolated VSMCs. Representative confocal IF images showing the basal, middle and apical surfaces of isolated VSMCs stained for F-actin (red), SUN1/SUN2 (green) and DAPI (blue).

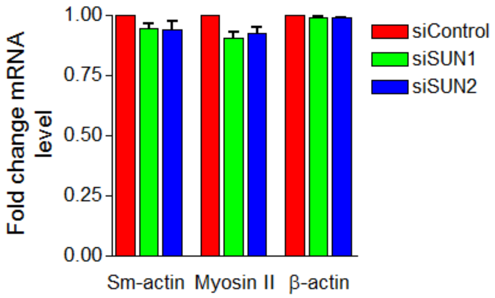

Supplementary Figure S3. QPCR analysis of sm-actin, myosin II and β-actin mRNA level in control, SUN1 and SUN2 depleted VSMCs. Graph represents the combined data of 3-independent experiments repeated in triplicate.

A. **B.**

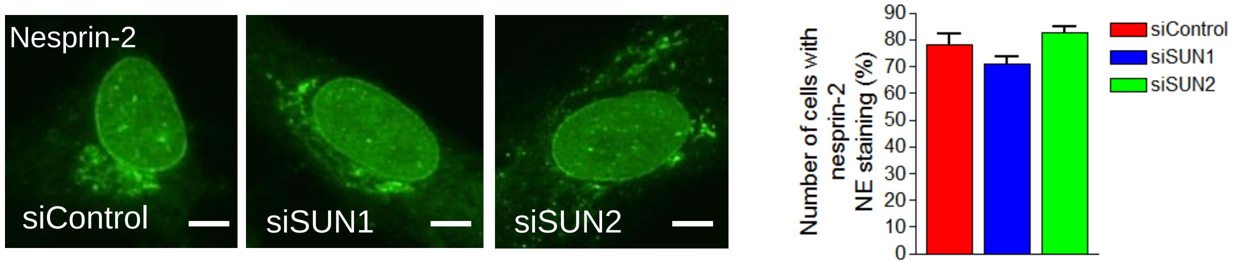

Supplementary Figure S4. Nesprin-2 remains localised to the NE in SUN1 and SUN2 depleted VSMCs. (A) Representative images of control, SUN1 and SUN2 depleted VSMCs stained for nesprin-2 (green). Scale bar represents 5µm. (B) Graph shows percentage of control, SUN1 and SUN2 depleted VSMCs displaying NE localised nesprin-2 and represents the combined data from 3-independent experiments analysing <300 cells.

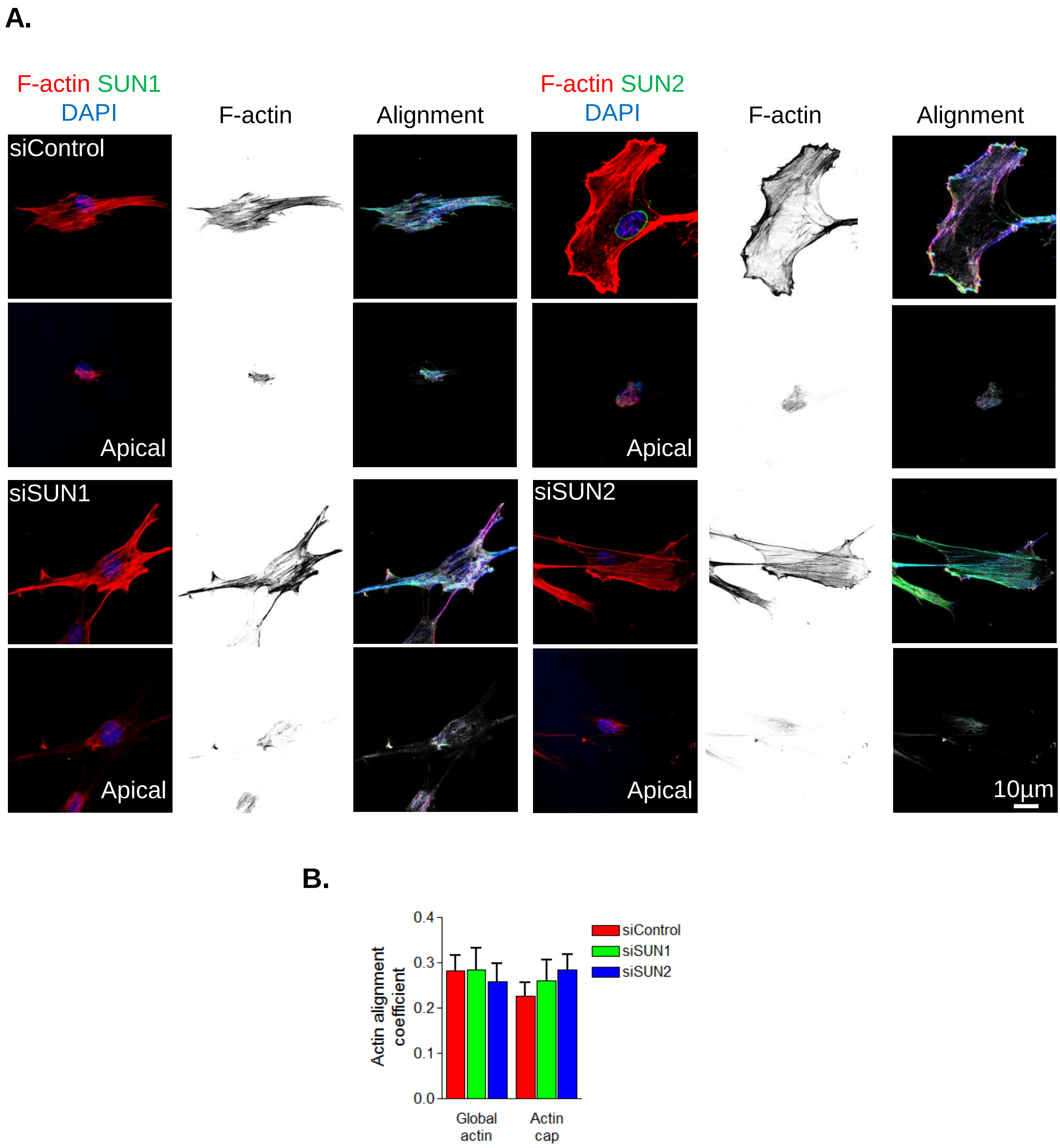

Supplementary Figure S5. SUN1 or SUN2 depletion does not alter F-actin alignment in isolated VSMCs. (A) Confocal IF analysis imaging the apical and middle sections of F-actin (red), SUN1 or SUN2 (green) and DAPI (blue) stained control, SUN1 and SUN2 depleted VSMCs grown on 12kPa hydrogels. These images represent the full cell images used in Figures 1C and D. (B) Graph shows the actin alignment coefficient of global actin and actin cap filaments Graph represents the combined data from 3-independent experiments analysing >20 cells.

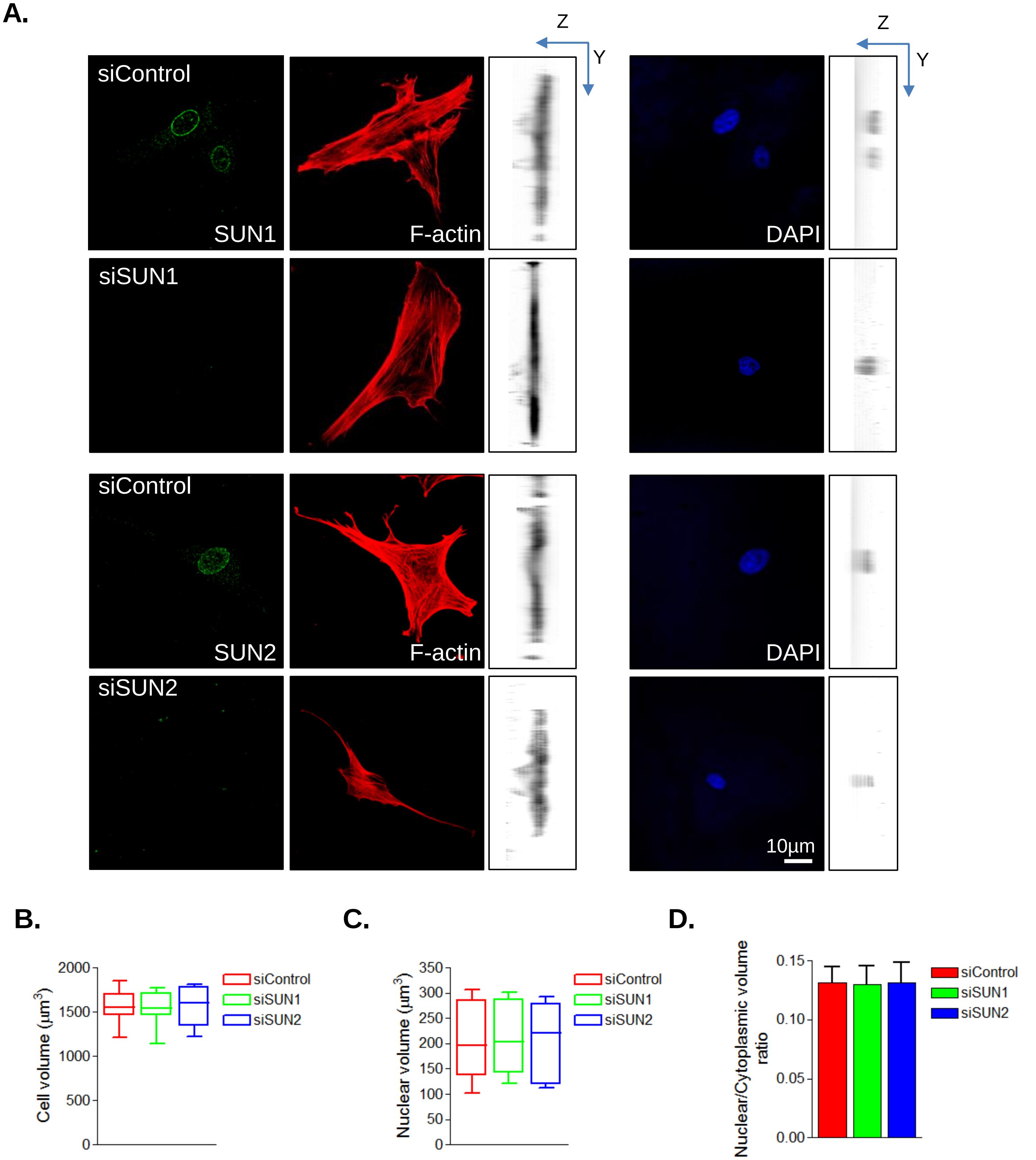

Supplementary Figure S6. SUN1 and SUN2 do not influence isolated VSMC volume. (A) Representative images showing the combined confocal stack of control, SUN1 and SUN2 depleted VSMCs grown on 12kPa hydrogels. Graphs show (B) cell volume, (C) nuclear volume and (D) nuclear volume/ cytoplasmic volume ratio of control, SUN1 and SUN2 depleted VSMCs. Graphs represent the combined data of 3-independent experiments analysing >20 cells.

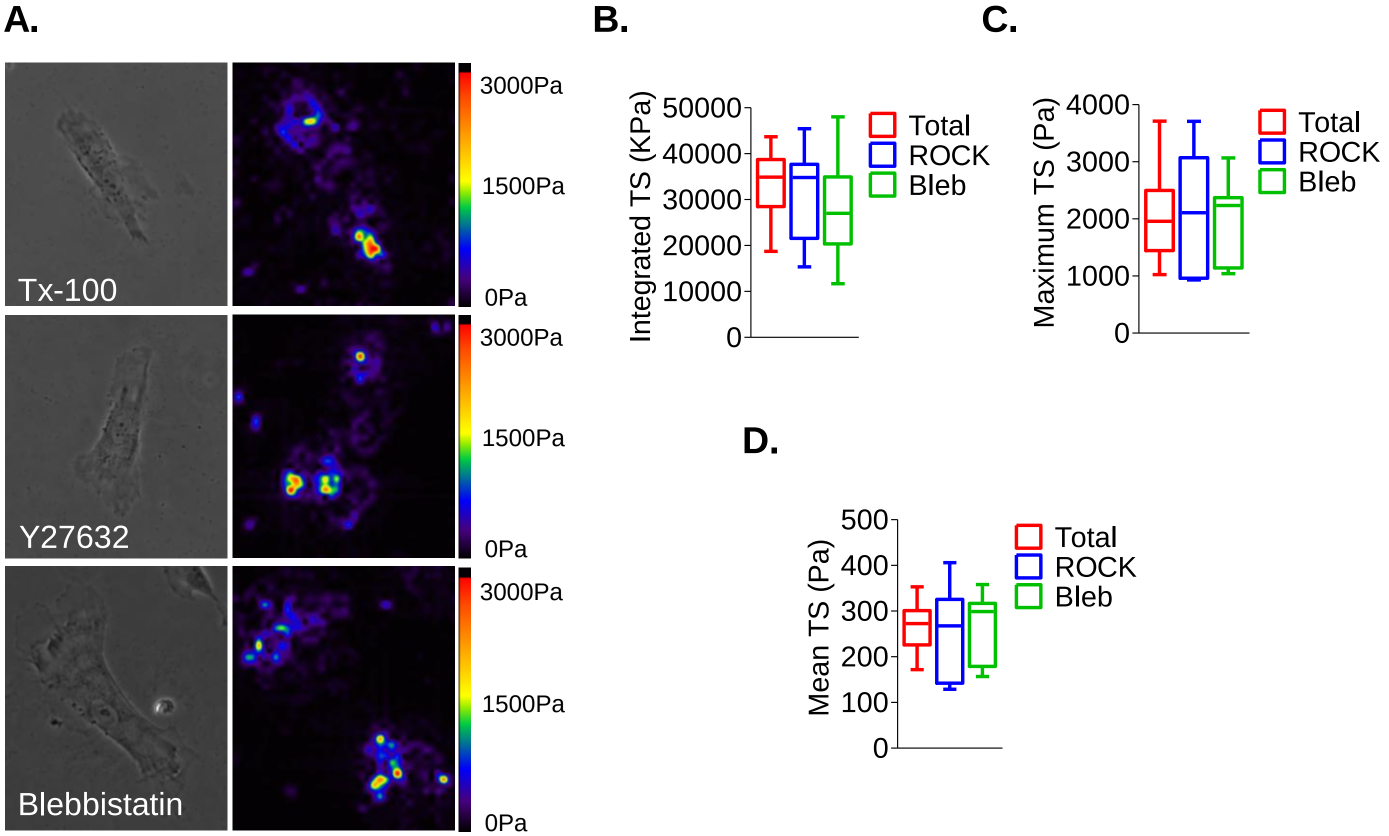

Supplementary Figure S7. Traction stress is regulated by a ROCK/myosin II pathway in isolated VSMCs. (A) Representative traction stress heat map of Tx-100 (total-TS), Y27632 (ROCK-TS) and blebbistatin (Bleb-TS) treated VSMCs. Graphs show (B) integrated-TS, (C) maximal-TS and (D) cell average-TS of control (n=18), Y27632 (n=19) and blebbistatin (n=24) treated isolated VSMCs. Graphs represent the combined data from 3 independent experiments.

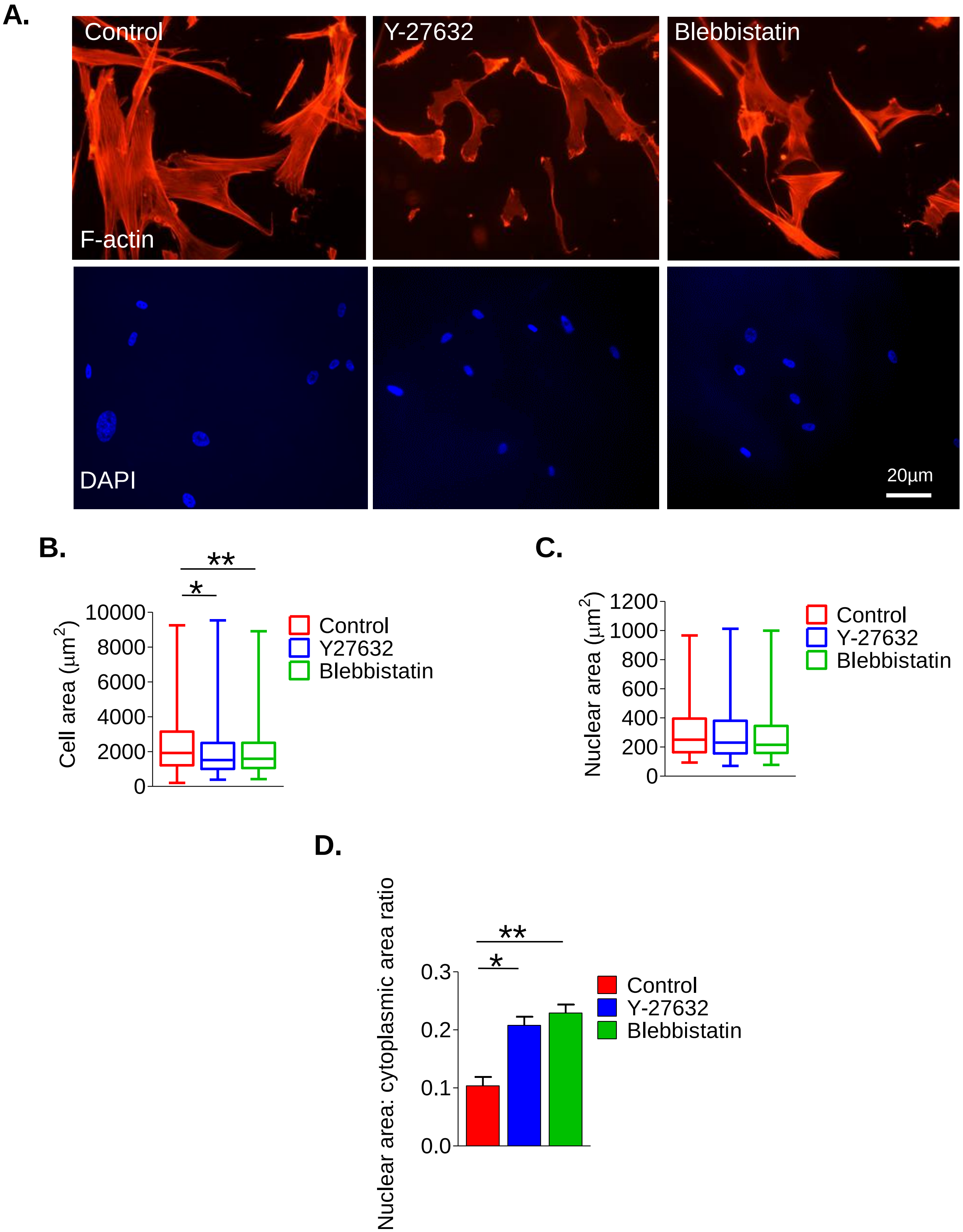

Supplementary Figure S8. VSMC area is regulated by a ROCK/myosin II pathway. (A) Representative images of control, Y27632 and blebbistatin treated VSMCs stained with Rhodamine phalloidin (red) and DAPI (blue). Graphs show (B) cell area, (C) nuclear area and (D) nuclear area: cell area ratio of control, Y27632 and blebbistatin treated VSMCs. Graphs represent the combined data from 3 independent experiments analysing >200 cells per group (*p= <0.05 and **p= <0.01).

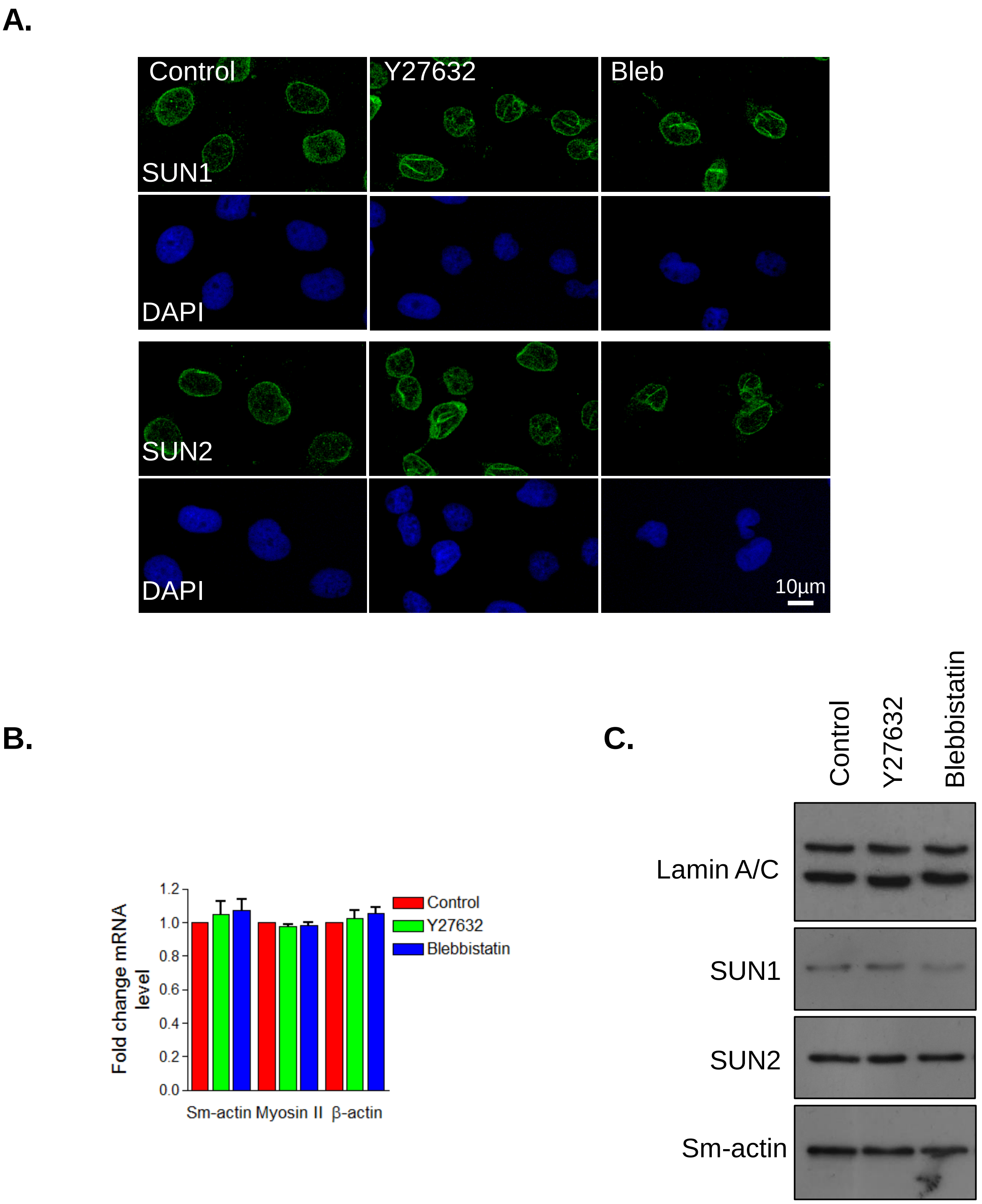

Supplementary Figure S9. Actomyosin activity does not affect SUN1/2 NE localisation. (A) Representative images of control, Y27632 and blebbistatin treated VSMCs stained for SUN1/2 (green) and DAPI (blue). (B) QPCR analysis of control, Y27632 and blebbistatin treated VSMCs. Graph represents combined data of 3-independent experiments repeated in triplicate. (C) WB analysis of control, Y27632 and blebbistatin treated VSMCs.

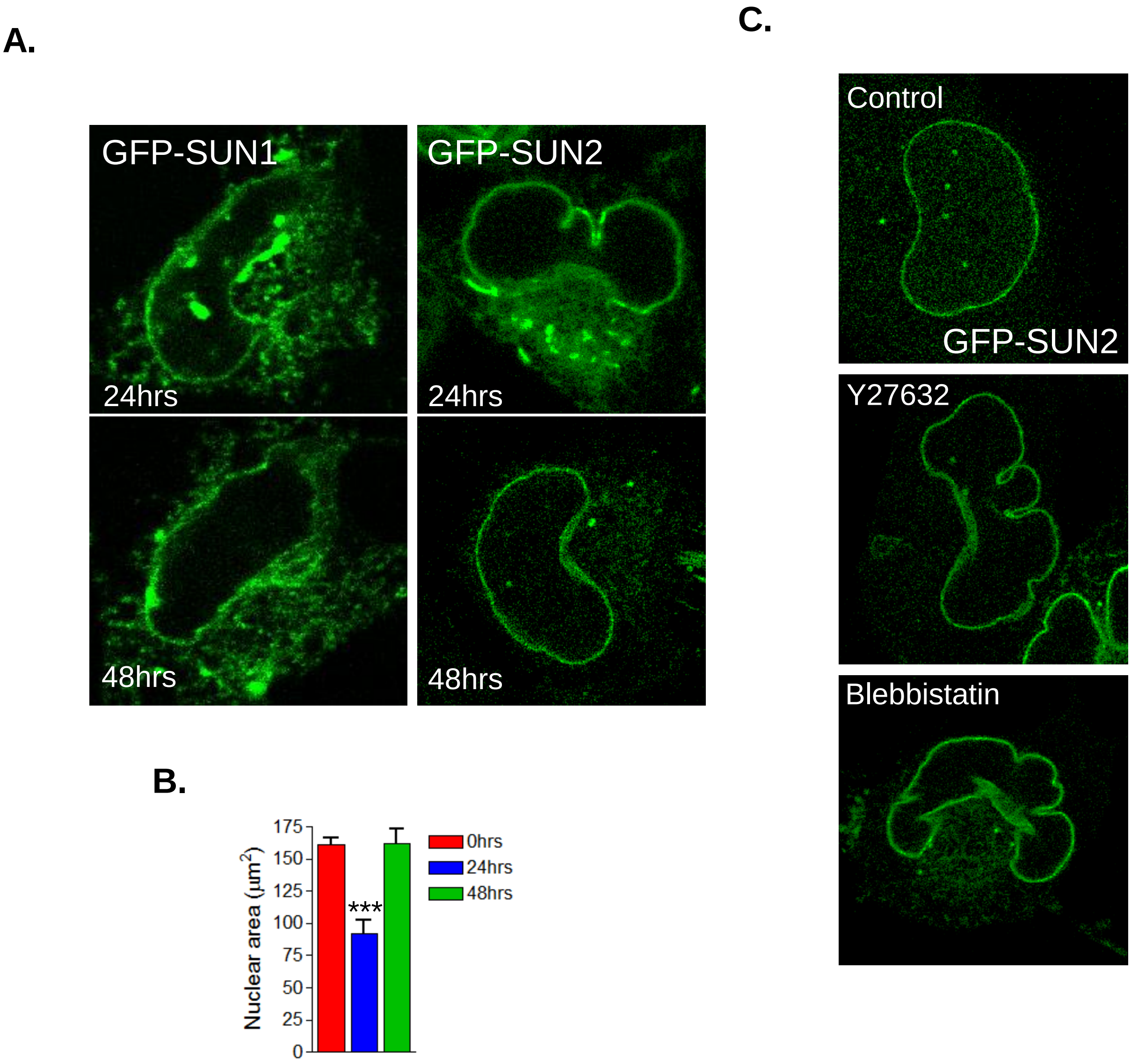

Supplementary Figure S10. SUN2 NE localisation is restored 48 hours after transfection. (A) Representative images showing GFP-SUN1 and GFP-SUN2 localisation in VSMCs 24 and 48hrs after transfection. (B) Graph shows nuclear area of control and GFP-SUN2 expressing VSMCs 24 and 48hrs after transfection and represents the combined data of 3-independent experiments analysing >100 cells (***p= <0.0001) . (C) Representative images of GFP-SUN2 localisation in control, Y27632 and blebbistatin treated VSMCs.
